# Supplementary figures and images for: Control of acute myeloid leukemia and generation of immune memory in vivo using AMV564, a bivalent bispecific CD33 x CD3 T cell engager
Source: PLoS One. 2024 May 2;19(5):e0300174. doi: 10.1371/journal.pone.0300174 (PMC11065199; doi:10.1371/journal.pone.0300174)

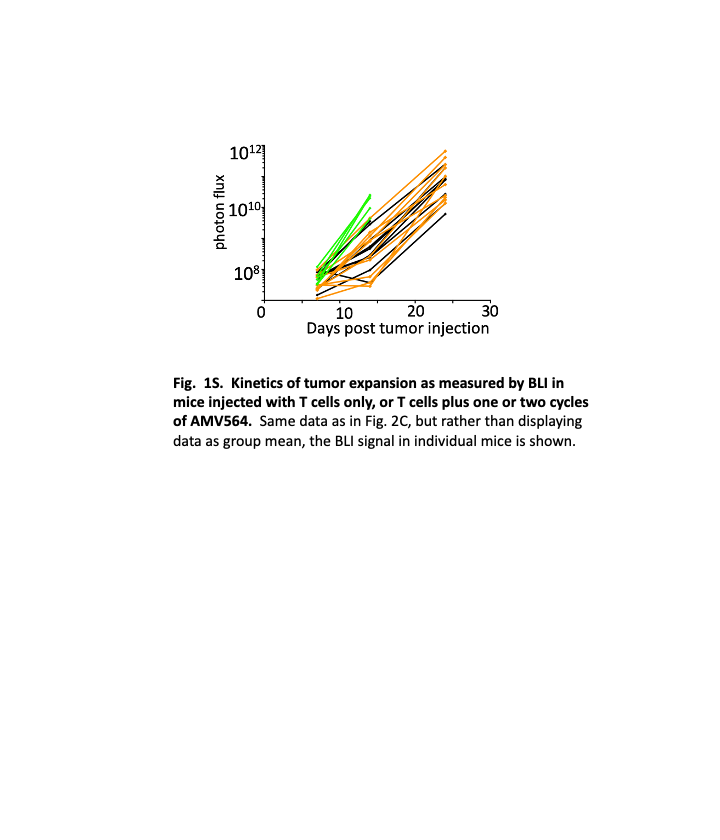

Supplement: S1 Fig — Same data as in Fig 2C, but rather than displaying data as group mean, the BLI signal in individual mice is shown. N = 5 mice/group for controls and n = 10 mice/group for mice treated with both T cells and AMV564. (TIFF) [file pone.0300174.s001.tiff]

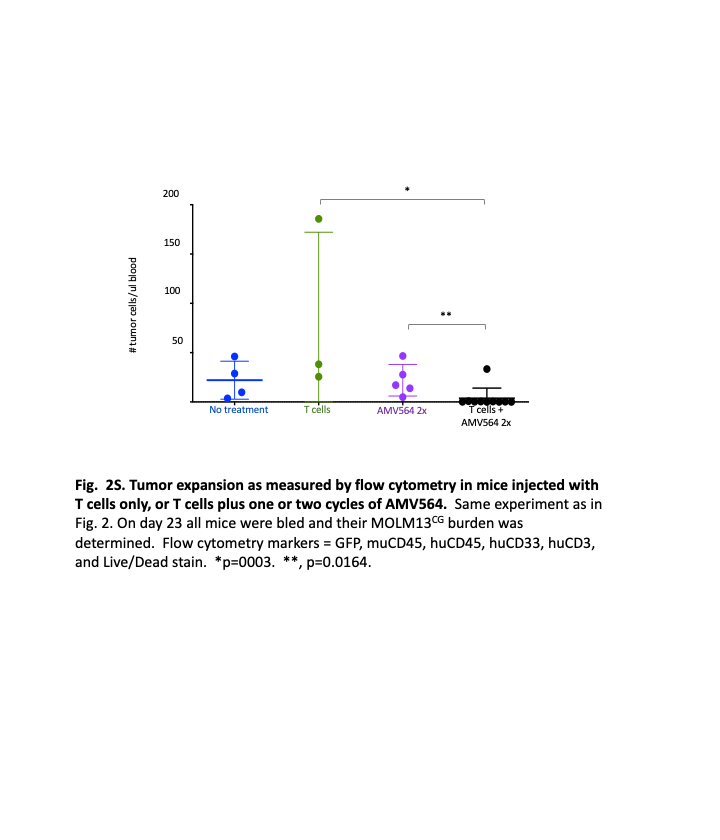

Supplement: S2 Fig — Same experiment as in Fig 2. On day 23 all mice were bled and their MOLM13CG burden was determined. Flow cytometry markers = GFP, muCD45, huCD45, huCD33, huCD3, and Live/Dead stain. *p = 0003. **, p = 0.0164. N = 5 mice/group for controls and n = 10 mice/group for mice treated with both T cells and AMV564. (TIFF) [file pone.0300174.s002.tiff]

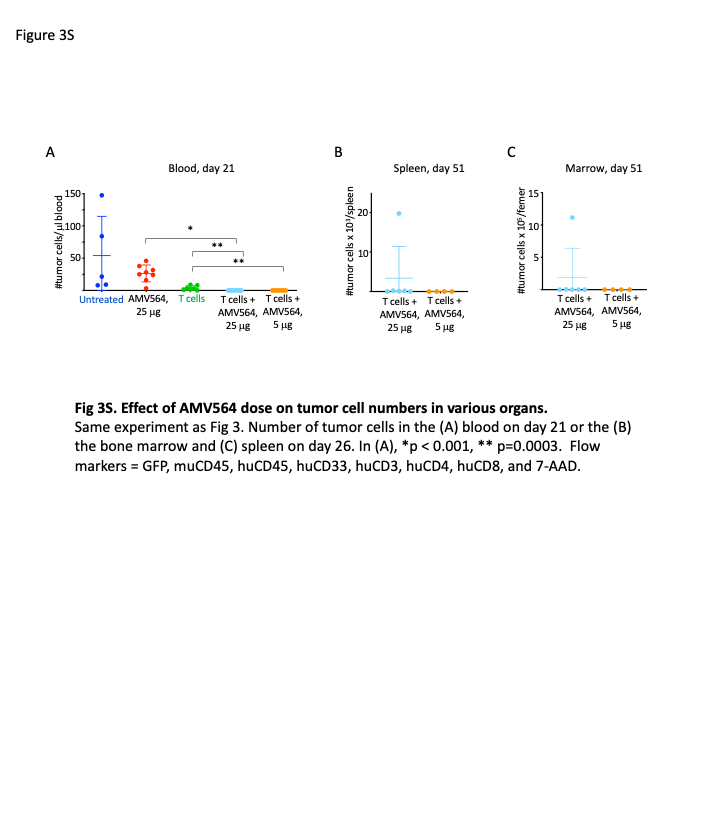

Supplement: S3 Fig — Same experiment as Fig 3. Number of tumor cells in the (A) blood on day 21 or the (B) the bone marrow and (C) spleen on day 26. In (A), *p < 0.001, ** p = 0.0003. Flow markers = GFP, muCD45, huCD45, huCD33, huCD3, huCD4, huCD8, and 7-AAD. N = 7 animals in untreated group. N = 8 animals in 25 mcg AMV group and in group treated only with T cells. N = 10 in groups treated with T cells in combination with either 5 or 25 mcg AMV564. (TIFF) [file pone.0300174.s003.tiff]

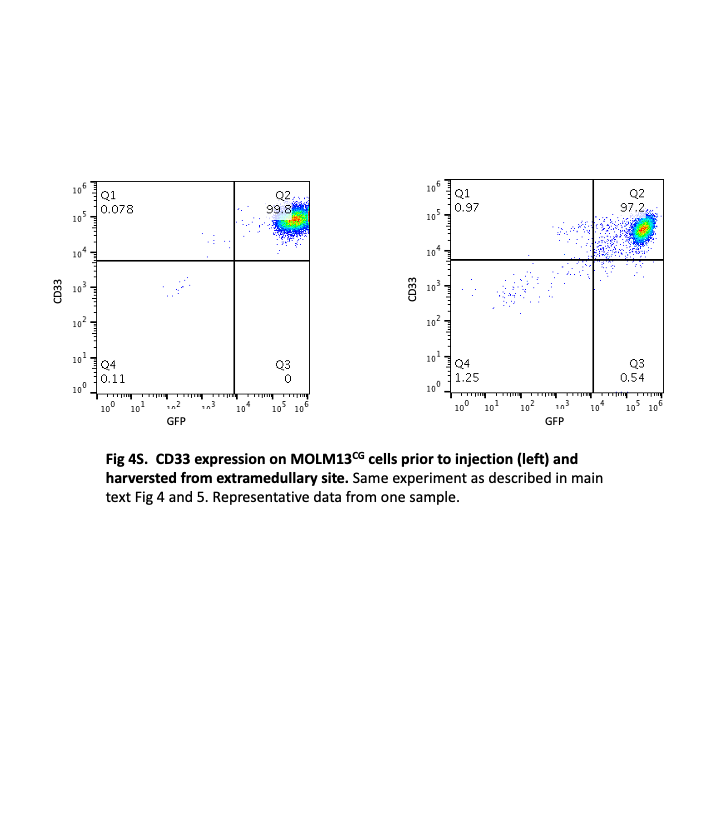

Supplement: S4 Fig — Same experiment as described in main text Fig 3. Representative data from one sample. N = 7 animals in untreated group. N = 8 animals in 25 mcg AMV group and in group treated only with T cells. N = 10 in groups treated with T cells in combination with either 5 or 25 mcg AMV564. (TIFF) [file pone.0300174.s004.tiff]

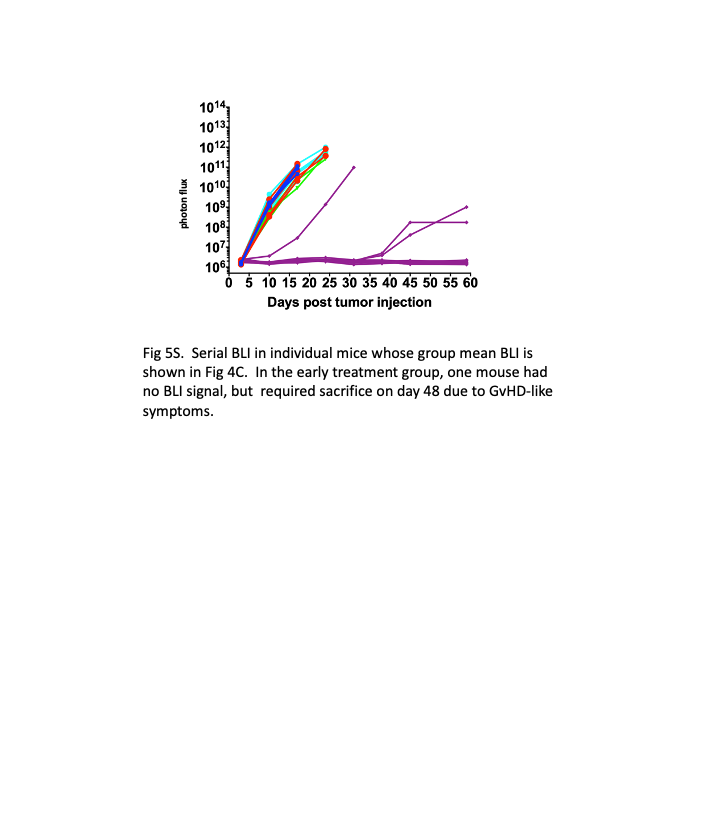

Supplement: S5 Fig — In the early treatment group, one mouse had no BLI signal, but required sacrifice on day 48 due to GvHD-like symptoms. For mice left untreated, treated only with T cells or only with AMV564, N = 5 mice/ group. For mice treated with both T cells and AMV564, N = 10 mice/group. (TIFF) [file pone.0300174.s005.tiff]

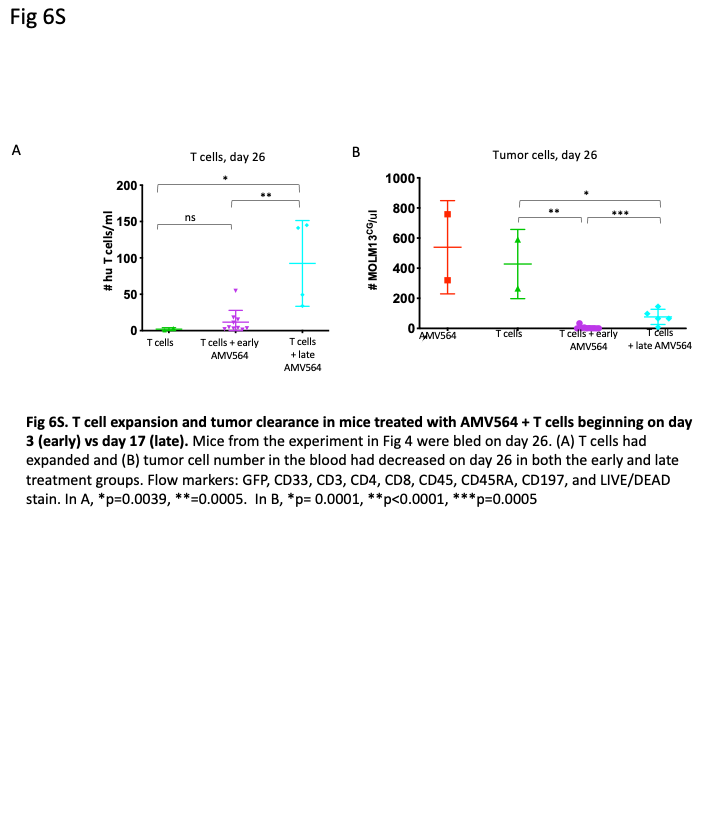

Supplement: S6 Fig — In A, *p = 0.0039, ** = 0.0005. In B, *p = 0.0001, **p<0.0001, ***p = 0.0005. For mice left untreated, treated only with T cells or only with AMV564, N = 5 mice/ group. For mice treated with both T cells and AMV564, N = 10 mice/group. (TIFF) [file pone.0300174.s006.tiff]
