# Supplementary material for: Control of acute myeloid leukemia and generation of immune memory in vivo using AMV564, a bivalent bispecific CD33 x CD3 T cell engager
Source: PLoS One. 2024 May 2;19(5):e0300174. doi: 10.1371/journal.pone.0300174 (PMC11065199; doi:10.1371/journal.pone.0300174)
Supplement: S1 Table — To assess moribundity, points are assigned to each of the 5 criteria below. A total score of 6 or higher or 30% weight loss, regardless of score, requires euthanasia of the animal. (DOCX) [file pone.0300174.s007.docx]

***Table 1S. Moribundity assessment of Animals.*** To assess moribundity, points are assigned to each of the 5 criteria below. A total score of 6 or higher or 30% weight loss, regardless of score, requires euthanasia of the animal.

|  | Score | | |
| --- | --- | --- | --- |
| Criterion | 0 | 1 | 2 |
| Weight | Normal* | Lost ≥10-25%* | Lost ≥25%* |
| Posture | Normal | Hunching only at rest | Severe hunching impairing movement |
| Activity | Normal | Mild to moderate decrease | Stationary unless stimulated |
| Fur texture | Normal | Mild to moderate ruffling | Severe ruffling/poor grooming |
| Skin integrity | Normal | Scaling of paws/tail | Obvious areas of denuded skin |

*Weight loss of 30% or evidence of hindlimb paralysis, regardless of total score, requires euthanasia.
