# Supplementary material for: Control of acute myeloid leukemia and generation of immune memory in vivo using AMV564, a bivalent bispecific CD33 x CD3 T cell engager
Source: PLoS One. 2024 May 2;19(5):e0300174. doi: 10.1371/journal.pone.0300174 (PMC11065199; doi:10.1371/journal.pone.0300174)
Supplement: S2 Table — (DOCX) [file pone.0300174.s008.docx]

**Table 2S. Details of animal usage in individual experiments, each performed once.**

|  | Duration (days) | Treatment groups | # mice/  group | # found dead,  i.e., died unexpectedly | #sacrificed due to hind limb paralysis, weight loss ≥ 30%, or tumor mass | #sacrificed due to moribundity score of 6^1^ | #sacrificed due to end of experiment |
| --- | --- | --- | --- | --- | --- | --- | --- |
| **Figure 2**  All mice were injected with tumor. | 42 | No treatment | 5 |  | 5 |  |  |
|  |  | T cells | 5 | 2 | 3 |  |  |
|  |  | AMV564 | 5 |  | 5 |  |  |
|  |  | T cells + 1 cycle AMV564 | 10 | 3 | 5 |  |  |
|  |  | T cells + 2 cycles of AMV564 | 10 |  | 10 |  |  |
| Total mice |  |  | 35 |  |  |  |  |
|  |  |  |  |  |  |  |  |
| **Figure 3**  All mice were injected with tumor.  Total mice | 51 | No treatment | 7 | 1 | 6 |  |  |
|  |  | T cells | 8 |  | 8 |  |  |
|  |  | 25 mcg AMV564 | 8 |  | 8 |  |  |
|  |  | T cells + 25mcg AMV564 | 10 |  | 5 |  | 5 |
|  |  | T cells + 5mcg AMV564 | 10 |  | 6 |  | 4 |
|  |  |  | 43 |  |  |  |  |
|  |  |  |  |  |  |  |  |
| **Figure 4**  All mice were injected with tumor . | 85 | No treatment | 5 | 1 | 4 |  |  |
|  |  | T cells | 5 |  | 5 |  |  |
|  |  | 5 mcg AMV564 | 5 |  | 3 | 2 blank |  |
|  |  | T cells + AMV564 on day 3 | 10 |  | 2 | 3 |  |
|  |  | T cells + 5mcg AMV564 on day 17 | 10 |  | 4 | 1 |  |
| Total mice |  |  | 35 |  |  |  |  |
|  |  |  |  |  |  |  |  |
| **Figure 5**  All mice were injected with tumor. | 62^2^ | Original challenge | 5^4^ | 1 | 2 |  |  |
|  | 115^3^ | Rechallenged mice | 2^4^ |  |  | 2 |  |
|  | 53^5^ | Untreated mice | 5 |  | 5 |  |  |
| Total mice |  |  | 10 |  |  |  |  |
|  |  |  |  |  |  |  |  |
| **Figure 6**  All mice were injected with patient PBMC.^6^ | 33 | No treatment | 8 |  |  |  | 8 |
|  |  | 5 mcg AMV564 | 8 |  |  |  | 8 |
|  |  | 50 mcg AMV564 | 8 |  |  |  | 8 |
| Total mice |  |  | 24 |  |  |  |  |

^1^Morbundity scoring is described in Table 1S.

^2^One group of 5 mice were injected with tumor and all were treated with both T cells and AMV564.

^3^All mice surviving on day 62 (i.e., 3 mice) were rechallenged with tumor cells.

^4^On day 63 one of the mice rechallenged with tumor cells the day before was found dead. A tumor was confirmed by BLI. Since it was unprecedented to have a mouse with a BLI detectable tumor 1 day after injecting MOLM13^CG^ cells, this tumor was presumed to have arisen from the original injection, and the mouse was eliminated from the rechallenge portion of the experiment. The death of this animal is recorded in the original challenge row as a mouse that was found dead.

^5^When the surviving mice in the above group were rechallenged with tumor cells on day 62, a second group of 5 previously untreated mice was injected with tumor for the first time, but received no treatment. The objective of this control was to verify that the tumor was progressing normally.

^6^This patient’s peripheral blood monocytes contained 98% tumor myeloblasts and 2% T cells.
