# Supplementary material for: Control of acute myeloid leukemia and generation of immune memory in vivo using AMV564, a bivalent bispecific CD33 x CD3 T cell engager
Source: PLoS One. 2024 May 2;19(5):e0300174. doi: 10.1371/journal.pone.0300174 (PMC11065199; doi:10.1371/journal.pone.0300174)
Supplement: Attachment — Submitted filename: Response to reviewers _11.28.23.docx [file pone.0300174.s011.docx]

**Cover letter statement to the Academic Editor, Benigno C. Valdez:**

We are grateful to all three reviewers for their time, thoughtful comments, and encouragement. Like both reviewers #1 and #3, we believe that the long-lasting immunity induced by treatment with the bispecific, AMV564, and shown in Fig. 5 is our most interesting finding. While we also agree that additional experiments would make this a more definitive conclusion, we deeply regret that due to insufficient resources, we are unable to move such work forward. However, since as pointed out by reviewer #1, our results, if substantiated and enlarged upon, have the potential to impact patient care using this or other bispecifcs. We believe the work, while limited, is sufficiently provocative that it needs to be shared with the scientific community in the hopes that others will see it as a fertile opportunity for additional investigation. PLOS ONE is the ideal venue for transmitting our findings since they are in keeping with the journal’s mission of evaluating research on its scientific validity, rigor, and high ethical standards with the stated aim of making all well-conducted research freely available.

We especially appreciate comments which led us to include a substantial amount of text in the abstract, results, and discussion that clarifies what we hypothesize is the mechanism behind the tumor control and protective immunity. In short, the bispecific is not only important for redirecting T cells to kill tumor cells independent of their innate T cell receptor (TCR), but also for providing a time window during which some T cells may become activated and proliferate via their normal TCR-dependent response. Once the concentration of the bispecific becomes negligible due to the brief 5-day treatment and short 18-23 h half-life, the tumor burden has shrunk so that the effector: target ratio has shifted and these naturally responding T cells assume responsibility for establishing and maintaining tumor suppression and ultimately establish a protective immune state. The possibility that remnant recalcitrant extramedullary tumor cells, may provide a low-level renewable source of antigen stimulation that aids in the persistence of the memory cells is discussed. As the reviewers point out, allogeneic responses (and possibly epitope spreading) may contribute to the results that we describe. We believe that the clarification and discussion of this hypothesis has strengthened the manuscript and its potential to impact translation of our findings, and we appreciate the opportunity to include it.

We removed any reference to cytokine release syndrome (CRS) in response to comments from all three reviewers.

We also removed the funding statement from the Acknowledgements as requested and have no changes to the current Funding Statement in the online submission form.

We request that our Competing Interests statement be updated to include the following: "This does not alter our adherence to PLOS ONE policies on sharing data and materials.”

*All line numbers refer to the clean version of the revised manuscript.* A tracked version will also be submitted. To better clarify and emphasize our hypothesis about mechanism, we substantially reorganized the discussion. Major changes are summarized in the table below.

| **Text beginning with this:** | **Was moved from:** | **To:** |
| --- | --- | --- |
| While our study was small | Sentence from fourth paragraph of discussion | First sentence of new first paragraph of discussion |
| The fact that memory T cells | (New paragraph) | Fourth paragraph of discussion |
| There are important distinctions | First paragraph of discussion | Sixth paragraph of discussion |

**Response to Journal Requirements:**

1. **Please ensure that your manuscript meets PLOS ONE's style requirements, including those for file naming. The PLOS ONE style templates can be found at**
   [**https://journals.plos.org/plosone/s/file?id=wjVg/PLOSOne_formatting_sample_main_body.pdf**](https://journals.plos.org/plosone/s/file?id=wjVg/PLOSOne_formatting_sample_main_body.pdf) **and** [**https://journals.plos.org/plosone/s/file?id=ba62/PLOSOne_formatting_sample_title_authors_affiliations.pdf**](https://journals.plos.org/plosone/s/file?id=ba62/PLOSOne_formatting_sample_title_authors_affiliations.pdf)

The manuscript has been re-formatted according to the style templates.

**2. Please provide additional details regarding participant consent. In the ethics statement in the Methods and online submission information, please ensure that you have specified what type you obtained (for instance, written or verbal, and if verbal, how it was documented and witnessed). If your study included minors, state whether you obtained consent from parents or guardians. If the need for consent was waived by the ethics committee, please include this information.**

Patients gave written consent as was stated in the “Cells and Cell Lines” subsection of the Methods in the original version. This has been moved to a new “Ethics statement” subsection as requested, lines 80-84.

**3. Thank you for stating the following in the Acknowledgments Section of your manuscript: 
"The work was supported by NIH/NCI: R35 CA210084 (JFD), NIH/NCI: P50 CA171963 NCI (JFD) and R50 CA211466-06 (MPR).  The authors also thank Dr. Joel C. Eissenberg for reading and editing the manuscript."**

**We note that you have provided funding information. However, funding information should not appear in the Acknowledgments section or other areas of your manuscript. We will only publish funding information present in the Funding Statement section of the online submission form. 
Please remove any funding-related text from the manuscript and let us know how you would like to update your Funding Statement. Currently, your Funding Statement reads as follows: 
"The work was supported by National Institutes of Health (NIH)/National Cancer Institute (NCI): R35 CA210084 (JFD), NIH/NCI: P50 CA171963 NCI (JFD) and NIH/NCI R50 CA211466-06 (MPR)."**

**Please include your amended statements within your cover letter; we will change the online submission form on your behalf.**

We removed the funding statement from the Acknowledgements and have no changes to the current Funding Statement in the online submission form. We have stated this in our cover letter.

**4. Thank you for stating the following in the Competing Interests section: 
"I have read the journal's policy and the authors of this manuscript have the following competing interests: THH and VS employed by Amphivena during this work; THH is currently an employee of ProfoundBio."**

**Please confirm that this does not alter your adherence to all PLOS ONE policies on sharing data and materials, by including the following statement: ""This does not alter our adherence to PLOS ONE policies on sharing data and materials.” (as detailed online in our guide for authors** [**http://journals.plos.org/plosone/s/competing-interests**](http://journals.plos.org/plosone/s/competing-interests)**).  If there are restrictions on sharing of data and/or materials, please state these. Please note that we cannot proceed with consideration of your article until this information has been declared.**

**Please include your updated Competing Interests statement in your cover letter; we will change the online submission form on your behalf.**

In our cover letter we have requested that our Competing Interests statement be updated to include the following: "This does not alter our adherence to PLOS ONE policies on sharing data and materials.”

**5. Please include captions for your Supporting Information files at the end of your manuscript, and update any in-text citations to match accordingly. Please see our Supporting Information guidelines for more information:** [**http://journals.plos.org/plosone/s/supporting-information**](http://journals.plos.org/plosone/s/supporting-information).

We have copied the captions of the supplementary figures to the end of the Manuscript file in addition to leaving them within the supplementary figures themselves.

**Response to the summary comments to the authors:**

**1. Is the manuscript technically sound, and do the data support the conclusions?**

We believe that the experiments shown were well designed and technically sound. Likewise, the conclusions reached were justified within the limitations of our available resources. We have attempted to be transparent within the text about where these limitations exist. A case in point is in lines 388-392 that indicate that in the rechallenge experiment in Fig. 5 which was designed to investigate the possibility of protective immunity, we were only able to start with 5 mice, and only 2 survived to day 62 for rechallenge.

2**. Has the statistical analysis been performed appropriately and rigorously?**

We acknowledged that each *in vivo* experiment was done only once. However, the experiments in figures 3, 4, and the initial experiment in figure 5 each built upon the previous experiments. Thus, they were essentially repeats in the sense that the observation and conclusions made in each of the predecessors needed to be valid in order to obtain the achieved results. In experiments 2, 3, and 4, all groups treated with AMV564 +T cells had 10 mice. Our conclusions are in keeping with the p values in these experiments. Unfortunately, we were only able to begin the experiment in Fig 5 with 5 mice in the AMV564+T cells arm. Of them, only 2 mice survived to be moved into the rechallenge experiment. The likelihood that either mouse would survive as long as, or a few days beyond, the never-treated control mice in the absence of additional AMV564, seemed low to us as is now stated in lines 402-404. Yet both mice survived for ~53 more days, nearly twice as long as the never-treated mice. This surprising outcome provides additional confidence that our conclusion that there is protective immunity is valid. In addition, it is buttressed by our finding that there were memory T cells circulating within the blood the day before rechallenge, thus providing a mechanism for survival. We regret that there are insufficient resources to repeat the experiment.

**3. Have the authors made all data underlying the findings in their manuscript fully available?**

All our data is available within the manuscript, figures, and supplemental figures. There is no large dataset that needs to be made available.

**4. Is the manuscript presented in an intelligible fashion and written in standard English?**

We agree with the reviewers that the manuscript was well written and intelligible. We have also had an expert science writer review it.

**Responses to individual reviewers comments:**

**Reviewer #1: In this manuscript, the authors test a bivalent CD3 x CD33 T cell engager in an AML model in vivo. The engager AMV564 can activate and expand T cells and suppress AML in vivo. In a few animals, this resulted in memory when the animals were rechallenged. An animal experiment with a primary AML and 2% T cells also suppressed AML with AMV564. This may be of translational value when considering AMV564 in the clinic.

The main critique of the manuscript is the limited data set, small number of experiments (n=1 in most cases and only 2 animals showing the memory response) leaving some questions about the definitiveness of the findings. Specific details are listed below.**

We are grateful to all three reviewers for their time and positive comments. We are pleased that both reviewers #1 and #3 believe, as we do, that the long-lasting immunity induced by treatment with the bispecific AMV564 and shown in Fig. 5 is our most interesting finding. While we also agree that additional experiments with more animals would make this a more definitive conclusion, we regret that due to insufficient resources we are unable to move such work forward. However, since like reviewer #1, we feel that our results have the potential to be translated into patient care using this and possibly other bispecifics and they need to be shared with the scientific community.

We attempted to be transparent about our data limitations, for example in lines 388-392 that indicate that in the rechallenge experiment in Fig. 5 which was designed to investigate the possibility of protective immunity, we were only able to start with 5 mice, and only 2 survived to day 62 for rechallenge.

We very much appreciate comments which led us to add a new first paragraph to the discussion, lines 453-473, regarding how tumor burden, E:T ratio, allogeneic responses, and epitope spreading might contribute to the mechanism behind tumor control and resistance to rechallenge. This is also referred to in the Abstract, lines 28-32, and in the Results, lines 263-269, and 350-358. We believe this addition has strengthened the manuscript.

**1) Figure 2 contains one experiment (how many animals should be in all legends) presumable from one normal donor T cell product. It makes it difficult to understand the reproducibility. While T cells + AVM564 prolong survival by 10 days, it is hard to completely understand the importance of this finding since all the animals still die, just 10-12 days later. Even with this finding, it would be good to known whether the CD33 targeting was important versus some non-specific activation. A control CD3 bivalent would address this issue. This is for the other experiments as well as resting T cells may be different than activated.**

The number of animals in each group was included in the figures themselves and has now also been added to all figure legends. We also added text to the results, lines 265-269, and the discussion sections, lines 463-466, indicating that we believe that in the experiment in Fig. 2, the bispecific initially redirected T cells to temporarily reduce the tumor burden. However, once the treatment was stopped and circulating drug became minimal or negligible, the effector to target ratio (E:T) was too low to control the tumor. In all later experiments a larger number of T cells was injected, thus increasing the in vivo E:T ratio once the drug was depleted and resulting in improved animal survival. This is likely due to the activation and expansion of allogeneic T cells while the drug was present, as well as possible activation of cells that responded to the tumor through their innate T cell receptor recognition. Both sorts of cells could have been present at the time the drug was becoming depleted, and they continued to exert tumor control often over several weeks in many mice. As stated to the academic editor, we regret that we do not have the resources to repeat the suggested experiments with a control bispecific or activated T cells.

**2) The inability of T cells + AVM564 to control extramedullary tumor seems a major limitation.**

We believe that the reviewer is suggesting that extramedullary tumor cells could provide a reservoir of tumor cells to seed a relapse, and we agree. However, we note that the animals appeared to be healthy despite this, and that the two animals in the rechallenge experiment likely had extramedullary tumor as well, since every animal in our experiment in Fig. 3 did. Nonetheless the animals quickly overcame a rechallenge of tumor cells. We concede that it is possible that additional treatment with the bispecific might have been able to eliminate the extramedullary tumor. However, it is also possible that the extramedullary tumor provided a renewable source of antigen, perhaps at low level, that encouraged persistence of the T cells. We include the latter as a possibility in lines 502-504.

**3) In the deaths speculated to be from CRS, could this have been neurotoxicity? Is there any direct data to support this speculation regarding CRS?**

We agree that the possibility of CRS being a source of animal death is unnecessarily speculative. We revised it to simply state that death appeared to be due to a highly inflammatory T cell mediated disease such as is observed in xenogeneic GvHD in our published studies, lines 358-360, 369-371, and 469-472. We have removed any reference to CRS.

**4) The most interesting finding is the lasting immunity, but this needs additional data since only 2 animals (out of 5) exhibited this activity. It still seems possible that this is allogeneic rejection primed by the reagents and it is not clear that this is antigen specific immunity. Could this be seen from multiple donors or is it a limiting finding. The reproducibility of this finding needs more rigor to understand it. Was the tumor control after rechallenge antigen specific? MHC restricted?**

We agree that the long-lasting immunity after treatment with AMV564 and T cells is our most interesting finding and that it could be a result of a primed allogeneic rejection of the tumor cells. Comments from two reviewers on this point caused us to extensively revise the discussion to acknowledge the contribution of both allogeneic cells that became activated by AMV564 as well as cells that became activated in response to their innate responses to antigens via their T cell receptors. We believe this strengthened the clarity of the manuscript and we appreciate the opportunity to add the text.

Please see the above reply to reviewer #1’s main critique.

**5) Would have been nice to see a survival readout for figure 6 using the primary sample and more than one AML.**

We agree and regret that we were unable to do this.

**6) The figure quality was generally poor.**

We are including new figure files that were put through the Preflight Analysis and Conversion Engine (PACE) digital diagnostic tool to improve image quality.

**7) In the discussion, the superiority comparison with AMG330 seems speculative and with no data or comparison presented. Added data would be needed to include this.**

We revised the text to outline differences between our study design and outcomes from those using other CD3 and CD33 bispecifics so that it doesn’t imply superiority of our reagent. We also removed the drug name “AMG330.” See lines 525-526.

**Reviewer #2:**

**The manuscript described interesting findings on the use of CD3-CD33 bi-specific antibody. The experiments were well designed, and conclusions were supported with data. One minor point I wish the authors would have done is to show without speculation that the mice that died from the treatment were actually due to CRS.**

Thank you for your supportive comments. Please see reply to reviewer #1 question 3.

**Reviewer #3**

**AMV564 is a tetravalent bispecific tandem diabodies targeting CD33 and CD3, which is currently being evaluated to treat AML patients in clinical trials. Here, authors attempted to explore efficacy and durability of responses using both in vitro and in vivo system. As its in vitro and vivo activity against HL60 has been already reported in original inventors, current works are unfortunately only incremental to what’s known with this construct even revised extensively. In addition, data presented are not sufficient to support main findings “ generation of immune memory in vivo using AMV564”.**

**Major
One interesting finding of the presented work is that 2 surviving mice from prior treatment were protected when they were rechallenged with same tumor. It would be critical to repeat experiment designed to test this phenomenon, and to investigate the mechanism of action, is it from alloreactive or antigen specific T cells recognizing Molm13-CD33 ? would it be against CD33, or other antigens present on the Molm13-CD33 suggestive of antigen spread phenomenon?**

Please see reply to Reviewer #1, comment 4.

**Minor**

**1) Please move figure legend at the end of manuscripts**

We understand the difficulty the reviewer likely had in reading the text due to the unconventional placement of the legends, but we did not move them since instructions for inserting the figure captions at the PLOS ONE website stated, “Figure captions must be inserted in the text of the manuscript, immediately following the paragraph in which the figure is first cited (read order).” (<https://journals.plos.org/plosone/s/submission-guidelines>)

However, we did copy the captions for the supplementary figures to the bottom of the manuscript as requested by the editor in addition to leaving them within the figure files.

**2) Please indicate source of T cells – healthy donor vs patients to all experiments**

This is now included in lines 94, 99, 191, 232, 270, 324, 389, and 424.

**3) This article requires some degree of scientific English editing throughout manuscripts**

The resubmitted text was reviewed and edited by an expert in scientific writing.

**4) Figure 1. Did author assess traditional T cell activation marker such as CD69 or 4-1BB ? If so, please include data.**

We followed only CD25. Please refer to Bajnok, et al., 2017, Mediators Inflamm.

(<https://www.ncbi.nlm.nih.gov/pmc/articles/PMC5438859/#:~:text=CD25%20is%20the%20alpha%20chain,and%20resting%20memory%20T%20cells>) as the source of the following text:

CD25 is the alpha chain of the trimeric IL-2 receptor and considered to be the most prominent cellular activation marker. It is expressed constitutively on the surface of several subsets of peripheral blood lymphocytes, such as regulatory and resting memory T cells.  It is upregulated within 24 hours of stimulation of the TCR/CD3 complex and remains elevated for a few days [[6](https://www.ncbi.nlm.nih.gov/pmc/articles/PMC5438859/#B6), [10](https://www.ncbi.nlm.nih.gov/pmc/articles/PMC5438859/#B10)]. It plays a key role in responsiveness to IL-2 resulting in lymphocyte activation and further IL-2 production. Several cytokines released by monocytes and macrophages as well as other agents triggering T cell activation (such as oxidized LDL) are capable of inducing CD25 expression [[11](https://www.ncbi.nlm.nih.gov/pmc/articles/PMC5438859/#B11), [12](https://www.ncbi.nlm.nih.gov/pmc/articles/PMC5438859/#B12)]. CD122 is the β-chain of the IL-2 receptor, and upon expression, it further increases sensitivity of activated CD25+ T lymphocytes to IL-2. Together with the common γ-chain and CD25, it forms the high-affinity trimeric IL-2 receptor [[13](https://www.ncbi.nlm.nih.gov/pmc/articles/PMC5438859/#B13)].

**5) Figure 3. “(E) Number of T cells in the blood of all mice on day 21 in the (F) bone marrow and (G) spleen of surviving mice on day 51” but corresponding figure indicate # cells/ul. total number of T cells or blasts in bloods, spleen, and marrow are more relevant to interpret the effects of T cell number, if T cells were enumerated, please indicate how absolute T cell numbers were calculated.**

Thank you for catching this. For blood samples we report number of T cells or blasts/ul. For spleen and marrow, we report total number of cells/organ. We corrected the axes in the figure and added text to the methods (lines 167-169) to explain how this was done.

**6) line 264-266 “the additional T cells appeared to provide a more substantial impact on tumor elimination (Fig 3C) with some mice completely clearing their tumors based on BLI measurements (Fig 3D)” - It appears authors infused T cells once with AMV564, it does not indicate an experimental arm that received additional infusion of T cells. please clarify. Also, results from figure 3 would not support “Mouse survival is more dependent upon T cell number than on AMV564 dose” as authors did not compare different T cell dose infused to xenografted mice or Figure 3E to 3G did not show any significant changes in number of “ cells” – assuming these were T cells.**

T cells were infused only once, but more of them were infused in the exp in Fig. 3 than in the one in Fig. 2. We indicated this in lines 269-272

and also added “a single dose of” to line 282, to avoid confusion.

The survival and tumor clearance comparison are being made between Fig. 3 which used more T cells, but the same 5mcg dose as was used in Fig. 2. See new text in lines 269-272.

The reviewer is correct that on day 21 there was no significant change in number of T cells (labeled on y-axis) between mice treated with T cells and either dose of AMV564 in Fig. 3E. Figs 3F-G show that the dose of AMV564 made no difference in number of T cells in the spleen or bone marrow. Together Figs. 3E-G simply show that there were large numbers of T cells in these organs. Lines 298-299.

**7) Figure 4. Please indicate how T cells and Molm13 tumor cells were enumerated from peripheral blood, and organ. For 4E, please specify the unit. Value ranged from 1 to 2 – but Y-axis showed “#molm13 cells/organ.”**

We apologize for the error and are grateful to the reviewer for noticing it. The figure has been corrected and we have added the calculation used to quantify cells to the flow cytometry section of the methods, lines 167-169.

**8) Please present data supporting “line 286: Extramedullary tumor cells remain despite treatment with AMV564 + T cells”, which sites were investigated ?**

This is now included in lines 149-152, 308-310, and 504-505. Sites were skin, testes, epididymis, small intestine muscle, and unidentified soft tissues. Visible tumor masses and random skin samples from the backs of mice were histologically reviewed.

**9) “line 333-334: Nonetheless it is noteworthy that by day 26, just two days later, there were high numbers of T cells in the blood (Fig 6S,A) and a substantially decreased number of tumor cells (Fig 6S,B) Consequently it seems likely that these mice may have died due to a highly inflammatory state or cytokine release syndrome (CRS) mediated by AMV564., ” I would suggest to move F6S to main body figure 4 as those are relevant information, and please present data to support possible CRS in mice with late treatment with high tumor burden if possible.**

We agree that this is relevant information and moved Fig. 6S into Figure 4 as panels F and G. We appreciate the reviewer’s suggestion.

We removed all reference to CRS in the revised manuscript. See reply to reviewer #1, question 3.

**10) Result line 349 “AMV564 can induce protective immunity in vivo: these results have driven from two mice who survived from initial treatment in single experiment. In order to conclude and investigate mechanism of protective immunity, experiments should be re-designed and performed to support above conclusion.**

We are deeply disappointed that we do not have the resources to repeat or redesign the experiments.

**11) Figure 6 – line 410-411: “The complete clearance or primary AML from all organs was accompanied by an expansion of T cells in vivo” suggest to revise conclusion – there was no differences in composition or absolute number of CD4 or CD8 T cells were not significantly different among no treatment vs treated group.**

We appreciate the suggestion and have changed the sentence as can now be found in lines 436-439.
